# A Hybrid Analysis-Based Approach to Android Malware Family Classification

**DOI:** 10.3390/e23081009

**Published:** 2021-08-03

**Authors:** Chao Ding, Nurbol Luktarhan, Bei Lu, Wenhui Zhang

**Affiliations:** College of Information Science and Engineering, Xinjiang University, Urumqi 830046, China; dingchao@stu.xju.edu.cn (C.D.); lubei@stu.xju.edu.cn (B.L.); zwh@stu.xju.edu.cn (W.Z.)

**Keywords:** android malware, malware detection and family classification, machine learning, hybrid analysis, dynamic networking flow

## Abstract

With the popularity of Android, malware detection and family classification have also become a research focus. Many excellent methods have been proposed by previous authors, but static and dynamic analyses inevitably require complex processes. A hybrid analysis method for detecting Android malware and classifying malware families is presented in this paper, and is partially optimized for multiple-feature data. For static analysis, we use permissions and intent as static features and use three feature selection methods to form a subset of three candidate features. Compared with various models, including k-nearest neighbors and random forest, random forest is the best, with a detection rate of 95.04%, while the chi-square test is the best feature selection method. After using feature selection to explore the critical static features contained in this dataset, we analyzed a subset of important features to gain more insight into the malware. In a dynamic analysis based on network traffic, unlike those that focus on a one-way flow of traffic and work on HTTP protocols and transport layer protocols, we focused on sessions and retained protocol layers. The Res7LSTM model is then used to further classify the malicious and partially benign samples detected in the static detection. The experimental results show that our approach can not only work with fewer static features and guarantee sufficient accuracy, but also improve the detection rate of Android malware family classification from 71.48% in previous work to 99% when cutting the traffic in terms of the sessions and protocols of all layers.

## 1. Introduction

The growth in applications on the Android platform has become unstoppable with the proliferation of smartphones and the advent of 5G, the latest generation of communication technology. The Ericsson Mobility Report [1] indicates that the number of mobile subscribers worldwide is approximately 8 billion at present, and this number is expected to increase to 8.9 billion by the end of 2025, of which mobile broadband subscribers will account for 88% of the total. By the end of 2019, there were 5.5 billion smartphone subscribers worldwide, and the report predicts that, by 2025, the number of smartphone subscribers will account for 85% of all cell phone users, with this number expected to reach 7.5 billion. Android OS accounted for 84.8% of global smartphone shipments in 2020, according to Smartphone Market Share [2], and the agency also predicts that Android OS is expected to account for 85.7% by 2024. Based on these two reliable data sources, the vast majority of smartphones worldwide in 2020 were Android OS, and their growth will continue at a high rate.

The explosion of malicious mobile applications has followed the growth in the number of smartphone subscribers. Internet Security Threat Report 2019 [3] data show that, in 2018, Symantec blocked an average of 10,573 malicious mobile apps per day, with a total of 2.238 million new mobile malware variants and 230 new malware families. In the TOP MALICIOUS MOBILE APP CATEGORIES for 2018, Tools topped the list, accounting for 39% of malicious apps, with LifeStyle and Entertainment at 15% and 7%, respectively. The mobile malware family Malapp accounted for 29.7% of the year’s malware volume and was the most intercepted malware family, followed by Fakeapp at 9.1% and MalDownloader at 8.9%. In addition, the percentage of mobile apps using invasive advertising techniques has declined, from 30% in 2017 to 26% in 2018. The total number of malware infections on mobile devices also declined in 2018, although the number of ransomware infections increased rapidly, by approximately 33% compared to 2017. The United States was the country most affected by mobile ransomware, accounting for 63 percent of all infections, followed by China with 13 percent and Germany with 10 percent. Securing mobile devices can be very challenging for organizations. During 2018, one in 36 devices used in an organization were classified as high-risk, including those with a high degree of certainty of being installed with malware.

This is even more serious, as shown in the 2019 Android Malware Special Report [4] released by the 360 Security Brain. The agency intercepted approximately 1.809 million new malware samples on mobile devices in 2019, of which fee consumption was the main type of new malware on mobile devices throughout 2019, totaling 847,000, or 46.8%, followed by privacy theft at 41.9% and remote control at 5.0%. In the third quarter of 2019, more than a quarter (26%) of in-app ads on Android worldwide contained fraudulent fake traffic content, with the highest rate of fake traffic being 33% for in-app programmatic ads registered in China [5]. On e-commerce platforms, media, advertising and other industries, there are fake traffic figures. The common methods of mobile traffic flow include malware infection, script-simulated clicks, tampering with API execution results, and cracking SDK code [4]. Therefore, it is crucial to detect Android malware and pay attention to malware family behavior at the same time. For more effective Android malware detection and family classification, this paper makes the following contributions to Android malware detection and family classification:

- The static detection of Android malware based on permissions and intents has been improved. We applied three feature selection methods, which eliminated more redundant features; a subset of candidate features was input to multiple machine learning methods, and the random forest was compared to yield the best detection results. The chi-square test is the optimal feature selection method and is briefly analyzed. Afterwards, we analyze the top 20 features in the optimal feature subset and one feature associated with them;
- The classification of Android malware families based on network traffic has been updated. In the dynamic analysis of Android malware, focusing on "sessions and all layers of protocols” in network traffic and applying Res7LSTM is considered feasible compared to focusing only on HTTPS in the application layer;
- Detection and classification processes are based on hybrid analysis. Permissions and intents are selected as static features and, after feature selection, different algorithms are applied to select the optimal algorithm and the optimal feature subset, and the results are directly output or input to the dynamic detection layer according to their prediction probability. In dynamic analysis, network traffic is split and imaged as “sessions and all layers of protocols”, and Res7LSTM is used for detection and classification.

This article is organized as follows: Section 2 discusses relevant work. Section 3 details our method of building the model. Section 4 introduces the experimental process and results. The conclusion and limitations of this paper is presented in Section 5.

## 2. Related Work

Android malware detection and family classification methods are divided into three main categories, namely, static analysis, dynamic analysis and hybrid analysis, and each of the three methods has its own advantages and shortcomings. Static analysis can guarantee a significant detection rate with less resource consumption than dynamic analysis, but static analysis does not capture the dynamic execution behavior of malware and is largely influenced by techniques such as obfuscation and packing. Hybrid analysis is a method that combines static and dynamic analysis but also requires as many resources and complex feature engineering as dynamic analysis. Static analysis mainly takes program components, permission declarations, etc., as detection features. Liu X and Liu J. [6] proposed a two-layer detection scheme to detect malware in Android applications based on the permissions contained in the manifest file. They requested permission pairs of applications as additional conditions for detection, and improve detection accuracy with the information contained in the used permissions, and the results show that the permission-based two-layer detector has a high malware detection rate. Although machine learning has been well-researched and explored in malware detection, this research is still lacking for ransomware detection. F. Noorbehbahani et al. [7] experimentally tested the validity of applying machine learning methods to ransomware detection in 2019, and demonstrated that random forests outperformed other tested classifiers on each experiment and that there was no significant change in the performance of the classifiers after different trainings on each classifier. Blanc W et al. [8] developed a lightweight static analysis method and defined a set of metrics for inspecting Android malware. Although the method achieves an FPR of 1.2% on the extracted features using random forests, it may still fail to identify code that has been obfuscated or shelled.

Dynamic analysis technology generally analyzes the characteristics generated when malicious software is dynamically executed, such as short messages or telephone numbers, API call sequences, and data access. The dynamic detection method obtains the behavior characteristics of application software by executing programs on sandboxes or real devices. Compared with static analysis, dynamic analysis is less affected by obfuscation and shelling, but it consumes resources and has difficulty covering all execution paths. GDroid [9] maps applications and their APIs to construct a heterogeneous graph, which performs better in both detection and family classification, but does not take into account the network behavior characteristics of malware, and therefore uses traffic files as a feature for dynamic analysis in our work. Describing malware as a two-dimensional image [10] is more convenient in feature engineering, which somewhat inspired our treatment when dealing with traffic files. [11] propose a feature fusion method, which uses both AlexNet and Inception-v3, and this method allows the model to extract different features from different aspects. [12] uses full connectivity and CNN for stacked ensembles. The stacked ensemble of these two different models is an effective method for complex traffic files.

Many types of malware have the ability to differentiate between environments, which makes dynamics-only analysis much less reliable. Hybrid analysis is an analysis technique that combines static and dynamic analysis to compensate for the shortcomings of static and dynamic analysis [13,14,15]. Lashkari A H et al. [16] proposed a systematic approach for generating Android malware datasets using real smartphones rather than emulators. In addition, 80 traffic features are used as the traffic feature set, and three classifiers—random forest, KNN and decision tree—are used to detect and classify malware families. Ref. [17] used random forests for both malware detection classification and malware family classification, with permission and intent as static features, and API calls and network traffic as dynamic features. Although the aforementioned studies have proposed many methods for malware analysis, some of them are less effective for multiclassification tasks such as family classification and category classification. First, we propose a hybrid-analysis-based multilevel approach to malware detection and family classification. This uses both a malware detection method based on static features such as permission and intent and a malware detection and family subclassification method based on mobile software network traffic. Second, we propose the Res7LSTM model with network traffic as the feature input as the second classification level. In the following sections, we discuss our model in more detail, and present our proposed approach from both methodological and experimental perspectives.

## 3. Modeling

**Figure 1:**
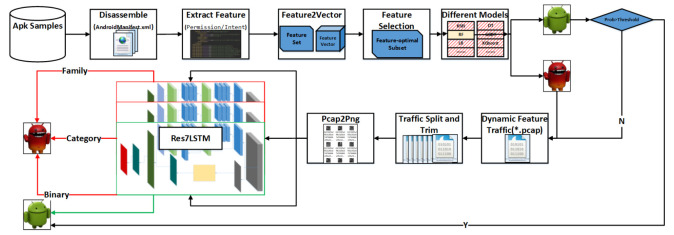
The framework of the model in this paper.

In this section, we propose a hybrid-analysis-based model for detecting Android malware and classifying malware families. Static detection is used as the static detection layer. First, static features are extracted, and then three feature selection methods, the chi-square test, analysis of variance (ANOVA) F-value, and mutual information, are used for feature selection [18]. Then, a 10-fold cross-validation was performed on the selected feature subset using machine learning methods such as KNN, multinomial NB, decision tree, random forest, SVC, NuSVC, LinearSVC, logistic regression, GBDT, and XGBoost [19]. Through experimental comparison, random forest was finally determined as the detection model for the static detection layer. In the second classification layer, dynamic analysis is used to extract features from the mobile traffic generated by the run, and then the Res7LSTM model is used to classify the software samples entered in the previous layer. The Res7LSTM model is also used to classify the malware into four major categories and 40 families. Figure 1 shows the overall structure of this paper.

### 3.1. Static Features-Manifest File Characterization

The static detection layer is a benign-malicious binary model with static features such as the permissions and intent of Android malicious code as input. Static features are extracted from the decompiled AndroidManifest.xml file, and then a subset of candidate features is obtained by feature selection. For optimal feature subsets and detection algorithms, the performance of various machine learning algorithm models on these feature subsets is compared. Although previous work [16,17,20] in this dataset is to be commended, little or no analysis of static features has been performed, leaving us with little insight into the behavior and intentions of the samples and the families they represent within the dataset. Therefore, we analyzed the static features of the dataset during the static detection process. The analysis of some of the features is shown in Section 4.4.

#### 3.1.1. Sample Decompiling and Obtaining Features

The Android malware samples used in this paper were obtained from the CICAndMal2017 dataset [16]. The samples were decompiled to obtain the files containing the features. The Apktool [21] tool was used to decompile all the apk samples to fetch *.smali, AndroidManifest.xml, etc., files for static analysis.

In [6], it is mentioned that the permission-based detection method is more suitable as a preliminary filtering mechanism for malware classification systems. The static features required by this method are acquired from the AndroidManifest.xml file, which contains information such as the permissions and intent of the apk sample application or use. By parsing the xml node data and namespaces in the AndroidManifest.xml file, we filter and obtain the permission and intent information it contains. As the attributes of the parsed node information will be changed by the namespace, the feature result is processed according to the value of the namespace “xmlns”, and 8111 static features are finally obtained.

#### 3.1.2. Initialize Static Feature Space and Obtain Numerical Feature Expression

The feature space of the dataset is obtained based on the features of all samples. On the basis of the feature space, the sample features are numerically expressed, and the frequency of the sample features is defined as their feature values. Before the feature vectors are used in the next step of detection, they should have preprocessing, such as normalization.

#### 3.1.3. Feature Selection and Mobile Malware Detection

Due to the large number of irrelevant and redundant features in the acquired feature vector, feature selection is necessary to analyze the static features and optimize the performance of the algorithm in a more reasonable way. We adopt the filter approach for feature selection to be independent of the detection algorithm. For more details, please see Algorithm 1. The three methods of feature selection, i.e., chi-square test, analysis of variance F-value, and mutual information, were used to obtain a subset of three candidate features. To select the optimal feature subset and the optimal detection model, the performance of different models on the three feature subsets is further compared. The optimal detection model is first determined based on the average performance of the different algorithmic models on the feature subset, and then the optimal feature subset is determined based on the performance of the selected optimal model on the different feature subsets. In this paper, it is experimentally concluded that the optimal detection model is random forest and that the optimal feature selection method is the chi-square test. We used scikit-learn [22], a machine learning library for Python, to implement the above methods. A more detailed feature analysis is provided in Section 4.4.
**Algorithm 1** Feature Selection.**Input:** Training set with permissions and intent static features;**Output:** Optimal subset of features after selection; 1  1:  **Step1:** Feature importance ranking  2:  The feature importance scores were calculated using chisquare test, analysis of variance Fvalue, and mutual information, respectively.;  3:  Removal of features with scores of Nan and 0;  4:  Obtain the corresponding candidate feature sets separately;  5:  **EndStep**  6:  **Step2:** Comparing the average performance of different algorithms  7:  Apply some detection algorithms to the three candidate feature sets;  8:  Calculate the average performance of each algorithm on the three feature sets;  9:  **EndStep**  10:  **Step3:** Obtain the optimal subset of features  11:  Compare the average performance and find the best-performing detection algorithm;  12:  Compare the performance of this optimal detection algorithm on three feature subsets and find the best performing feature set;  13:  **EndStep**  14: Return the optimal subset of features.

To further understand the behavioral intent of Android malware as opposed to benign software, we analyze and discuss a selection of the top 20 important features and an interesting feature related to them. These will be found in Table 1.

### 3.2. Dynamic Features—Mobile Network Traffic Data Mining

The inputs to this level are the dynamic traffic characteristics corresponding to the output samples from the static detection layer. Based on the benign–malicious probability of the output in the static detection layer and a predetermined threshold value, it is determined whether the second classification layer should be input, or whether the results should be directly output. If the output label of the previous layer is benign and its probability is equal to or greater than the threshold (in this paper, the threshold is set to 1), it can be confirmed as benign and the “benign” label can be directly output; if the probability of the benign label is less than the threshold, the sample will be input to the current classification layer for further detection and classification; if the output label of the previous layer is malicious, it can be directly input to the current classification layer for further detection and classification. The sample is input into the current classification layer to perform further classification. The second classification layer uses our proposed Res7LSTM model to perform classification tasks such as benign–malicious classification (two classification categories), malware class classification (four classification categories), and malware family classification (forty classification categories) on the dynamic network traffic characteristics of the input sample. The design of this method is as follows.

#### 3.2.1. Dataset

The dynamic network traffic characteristics used in this method are derived from the CICInvesAndMal2019 [17] dataset, the second part of the CICAndMal2017 [16] dataset. The dataset collected 10,854 samples (4354 malware and 6500 benign) from multiple sources, with the benign samples being collected from Google Play in 2015, 2016 and 2017. The dataset installs 5000 collected samples (426 malware and 5065 benign) on real devices and divides malware samples into four categories (adware, ransomware, scareware and SMS malware). A total of 426 of these malware were from 42 malware families. To overcome the stealthy nature of advanced malware, three data-capture states were defined (during installation, before reboot and after rebooting the phone) capturing all log files (including network traffic, battery state, log state, program packages, process logs, etc.) generated by the samples in a real smartphone environment connected to the network.

#### 3.2.2. Preprocessing

The granularity of network traffic splitting is TCP connection, traffic, session, service and host [23]. Different splitting methods yield different units of traffic. A session is a bidirectional flow and includes two-way traffic. A flow is defined as a set of contiguous packets with the same five tuples (source IP, source port, destination IP, destination port, and transport level protocol), and a session is a bidirectional flow in which the source and destination IPs are exchanged.

Our handling of network traffic is different from [20], which focuses more on the HTTP protocol and the UDP and TCP protocols in the transport layer and treats pcap file by filtering out the protocols of interest and separating them into the basic flow. We believe that, in general, a session contains more interactive information than a unidirectional flow. In addition, the all-layer representation of a packet contains more layers than the transport layer representation, especially including information that the transport layer does not contain, such as IP Header and Frame Number, so more critical information can be expressed. To preserve more vital information, we chose to slice the traffic file in terms of the session and all layer protocols.

(1) Labeling and Pcap2Session.
  To slice the traffic file, Wang et al. [24] proposed a new traffic classification method for malware traffic based on convolutional neural networks using traffic data as images. USTC-TK2016, the paper’s publicly available traffic handling tool, is based on SplitCap.exe and integrates applications such as finddupe.exe. When all layers are preserved, the data link header contains information about the physical link. This information, such as the media access control (MAC) address, is essential for forwarding frames over the network, but it has no information for application identification or traffic characterization tasks [25]. Since the MAC address changes when crossing domain gateways, causing inconsistency problems, we filter the source and destination addresses as traffic packets for MAC addresses. The traffic files are organized in a structure of “two-classification-labels/four-classification-category-labels/forty-classification-family-labels/network-traffic-files” (the four classification and forty classification labels for a benign sample are all None) to facilitate subsequent labeling. The resulting folder has the same name as the pcap file and maintains its folder organization.
(2) Pcap2Png and Png2Mnist.
  This step first divides the sliced and filtered mac address pcap files randomly into training and test sets at a ratio of 9:1. The divided pcap files are then unified to the same length. The work of Wang Wei et al. [24] shows that trimming the cut pcap file to 784 bytes leads to better results, and that dividing by session is better than dividing by flow. Therefore, the choice was made to slice by session with the the length = 784. The obtained pcap file in the previous step is converted to a single-channel grayscale png image with three kinds of label (benign–malicious labels, category labels and family labels). Three kinds of ubyte dataset file were constructed with reference to the Mnist dataset format [26].

#### 3.2.3. Dynamic Analysis, Training and Evaluation

The converted data in Mnist format were used as the input of the Res7LSTM model, and the labeled binary, four-category and forty-category data were used to train the Res7LSTM to obtain the three classification models. The performance evaluation metrics of the models include accuracy, precision, recall and F-measure.

### 3.3. Detection and Classification

The Res7LSTM model in this paper combines a residual network (ResNet) and a long short-term memory (LSTM) model as a second classification layer used for detection, category and family classification.To avoid overfitting, we used batchnormallization and DropOut [27] in the construction of the model. In addition, the combination of models using Res7LSTM also avoids overfitting to some extent.

#### 3.3.1. Residual Network

**Figure 2:**
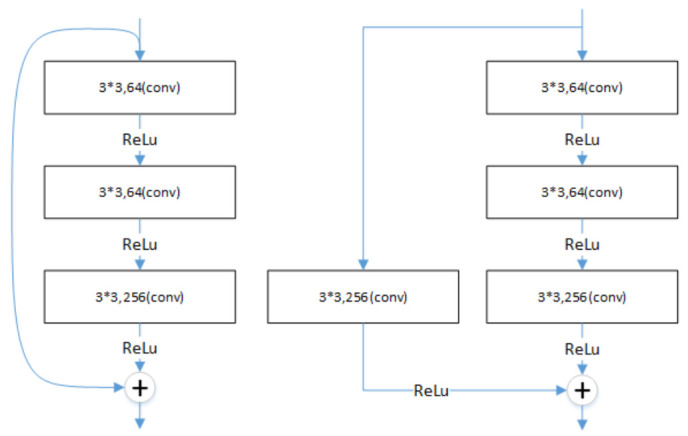
The connection of ResNet50.

The degradation problem occurs when the deeper networks start to converge: as the depth of the network increases, the accuracy saturates and then degrades rapidly. However, this degradation is not caused by overfitting, and adding more layers to a suitably deep model leads to more training errors [28,29]. ResNet (residual neural network) [30] proposed a deep residual learning framework to solve the degradation problem of deep networks. A connection method called “shortcut connection” is used in ResNet, which allows the network to retain a portion of the output of the previous layers. ResNet50 is a typical model of this type of network with two types of “shortcut connections”, as shown in Figure 2.

#### 3.3.2. Long Short-Term Memory

**Figure 3:**
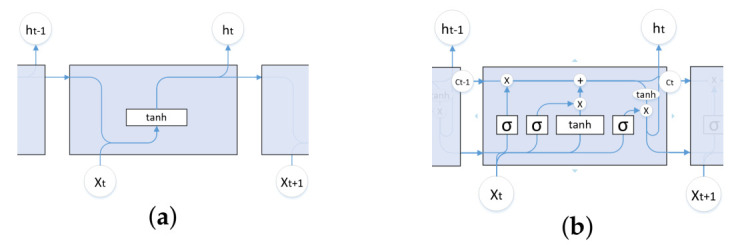
Basic units of RNN and LSTM. (**a**) Basic units of RNN. (**b**) Basic units of LSTM.

LSTM [31] is a special recurrent neural network (RNN) [32]. LSTM solves the long-term dependence problem in RNNs, i.e., gradient disappearance or explosion. LSTM differs from RNNs in their basic units. The basic structure of the cells of both models is shown in Figure 3. The advantage of LSTM is its ability to select and determine what information can be retained or ignored by the gate structure. There are three basic structures in each cell node of this network structure, which are input gates, forgetting gates, and output gates, as shown in Figure 3b.

#### 3.3.3. Malware Category and Family Classification

**Figure 4:**
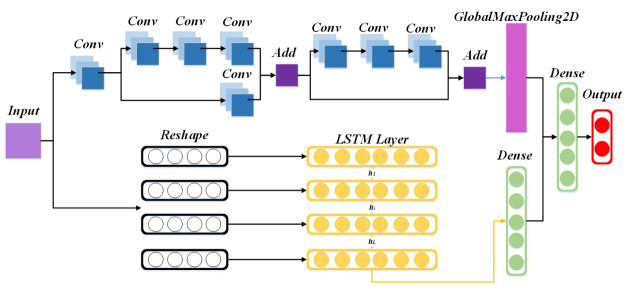
The Res7LSTM structure.

The Res7LSTM model in this paper is a combination of a residual network (ResNet) and long short-term memory (LSTM). Figure 4 depicts the network structure built by this model. The BatchNormalization layer, the activation layer, and some pooling layers are ignored for the sake of simplicity and clarity. As depicted in the figure, we constructed a neural network with seven layers of ResNet and an LSTM. First, the input goes through a residual network with seven layers of convolution, then a GlobalMaxPooling2D layer; meanwhile, the initial input goes through an LSTM network with 128 hidden neurons and a fully connected layer. The two vectors above are connected and input to a fully connected output layer with a softmax activation function. Res7LSTM contains a residual network, which has a convolution layer, a residual block with three convolution layers and no convolution layers in the side path, and a residual block with four convolution layers and one convolution layer in the side path, where the activation functions of the convolution layers are *ReLU*. Finally, the model’s optimizer is selected as *Adam*, and its learning rate is set to *0.01*.

## 4. Experimental

In this section, first, the dataset used is presented in Section 4.1. The data-processing scheme of this method is described in Section 4.2. Next, the performance metrics used in the evaluation of our experiments are presented in Section 4.3. In Section 4.4, the experiments of feature selection and the performance comparison of candidate models on a subset of features are described in detail, and the optimal model and the optimal subset of features are identified. Additionally, in this section, we provide a brief analysis of the validity of the chi-square test and the 20 features selected. In the final Section 4.5, our proposed method is compared with other methods.

### 4.1. Complete Dataset Description

We use data from the CICAndMal2017 [16] dataset and CICInvesAndMal2019 [17]. This dataset collected over 6000 benign apps from the Google Play Marketplace, of which 1435 were removed (leaving a total of 5065 apps) because they were flagged as suspicious or adware by more than two antivirus products in Virustotal [33]. The dataset collected 4354 malware items from multiple data sources. Due to “sample errors” and “inconsistent malware labels”, they installed and ran only 429 malware and 5065 benign software on real devices.

In the network traffic generated by the 42 malware families published in this dataset, two families are not included in the static detection used in the static detection layer, which are Koodous in Adware and FakeApp. AL in Scareware. To accurately evaluate our model and to ensure the consistency of the detection and classification layers, we finally chose to drop these two families based on the samples used in the static detection layer. This paper uses 1190 and 404 captured network traffic generated by benign and malicious samples, respectively. There are four categories of malware, namely, Adware, Ransomware, Scareware, and SMS; there are 40 malware families, and the distribution of families and datasets is shown in Table 2.

### 4.2. Static and Dynamic Feature Data Preprocessing

The static features used in this method were extracted from the AndroidManifest.xml file in the decompiled sample generation file. The dynamic features used for analysis are the network traffic that is captured while the mobile application is run dynamically. To apply deep learning methods and simplify feature engineering, we trimmed the network traffic to a fixed length (784 bytes) after slicing the file by session and converting it to grayscale images in png format. The total number of grayscale images after processing was 437,782 for benign samples and 749,190 for malicious samples; 217,598 for Adware and 196,803, 198,481 and 136,308 for Ransomware, Scareware and SMS, respectively.

### 4.3. Evaluation Metrics

The performance metrics used during the experiments will be presented in this subsection due to the requirements of selecting the optimal feature subset and comparing the performance of different algorithms in the static detection layer and other methods in the second classification layer.

The commonly used metrics for performance evaluation are precision, recall, F1-measure [34], and accuracy. Accuracy is the proportion of samples that are correctly classified to the total number of samples, calculated as in Equation (1). Precision is t heratio of samples that are predicted to be positive and are actually positive to the samples that are predicted to be positive, as calculated in Equation (2). Recall refers to the ratio of samples that are predicted to be positive and are actually positive to the samples that are actually positive, using Equation (3). Equation (4) represents the F-measure, which is the harmonic mean of the precision and recall rates, α=1 in this paper.

(1) $$accuracy=\frac{TP+TN}{TP+FP+FN+TN}$$

(2) $$precision=\frac{TP}{TP+FP}$$

(3) $$recall=\frac{TP}{TP+FN}$$

(4) $$f-measure=\frac{(\alpha^{2}+1)precision*recall}{\alpha^{2}\left(precision+recall\right)}$$

In the above equations, FP denotes the number of samples that were predicted to be positive but were actually negative, TN denotes the number of samples predicted to be negative and actually negative, TP denotes the number of samples predicted to be positive and actually positive, and FN denotes the number of samples predicted to be negative but actually positive. When evaluating the performance of multiple classifications, the common metrics were calculated in two ways: macroaverage and microaverage. Macro calculates the precision and recall on each confusion matrix, then calculates the average value, and finally calculates the F1-measure. Equations (5)–(7) were used for the calculations. Micro is the average of the corresponding elements of the confusion matrix, resulting in $\bar{TP}$,$\bar{FP}$,$\bar{TN}$,$\bar{FN}$, on the basis of which Micro-P, Micro-R, and Micro-F1 are calculated. Equations (8)–(10) were used for the calculations.

(5) $$Macro-P=\frac{1}{n}\sum_{i=1}^{n}precision_{i}$$

(6) $$Macro-R=\frac{1}{n}\sum_{i=1}^{n}recall_{i}$$

(7) $$Macro-F1=\frac{2*Macro-P*Macro-R}{Macro-P+Macro-R}$$

(8) $$Micro-P=\frac{\bar{TP}}{\bar{TP}+\bar{FP}}$$

(9) $$Micro-R=\frac{\bar{TP}}{\bar{TP}+\bar{FN}}$$

(10) $$Micro-F1=\frac{2*Micro-P*Micro-R}{Micro-P+Micro-R}$$

### 4.4. Feature Selection and Detection Algorithm Comparison

**Figure 5:**
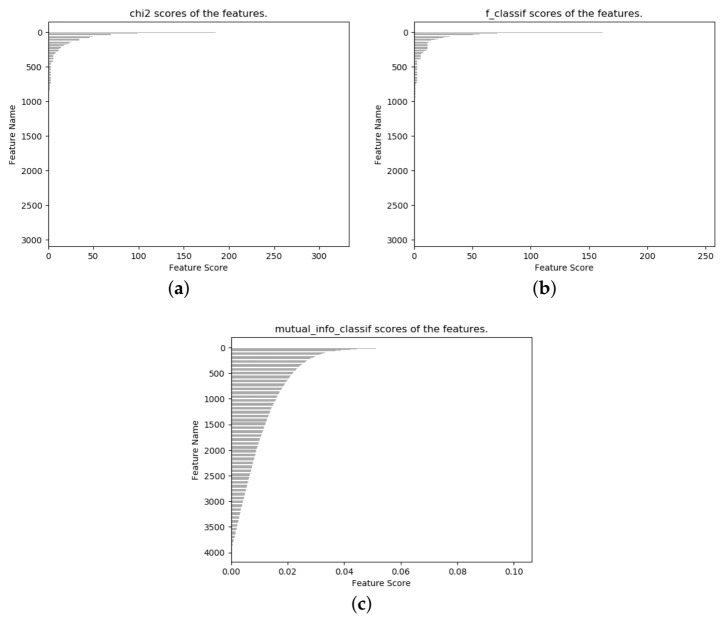
Feature selection. (**a**) chi-squared test. (**b**) ANOVA F-value.(**c**) mutual information.

The static features used in the static detection layer of this thesis had a total of 8111 features after numerical analysis. We used three feature-selection methods based on the chi-square test, analysis of variance F-value, and mutual information to rank the features in terms of feature significance, and the feature selection results are shown in Figure 5. After feature importance ranking, only 2949 features in Figure 5a,b had nonzero feature importance indicators, and 4031 features in Figure 5c had nonzero feature importance indicators. Finally, we determined the number of features to be 784 for the purpose of applying the model more broadly, and obtained three feature subsets: *chi2_784*, *f_784*, and *mutual_info_784*. Despite our expectation that the dimensions of the selected subset of features would be suitable for more deep learning models, the model we selected was so unsatisfactory that we abandoned the idea of using and comparing the selected deep learning models. Therefore, we hope to gradually improve this aspect in our future work, to find a suitable model and method. This is also the second reason that we chose 784 as the final number of features but did not compare the deep learning models.

**Figure 6:**
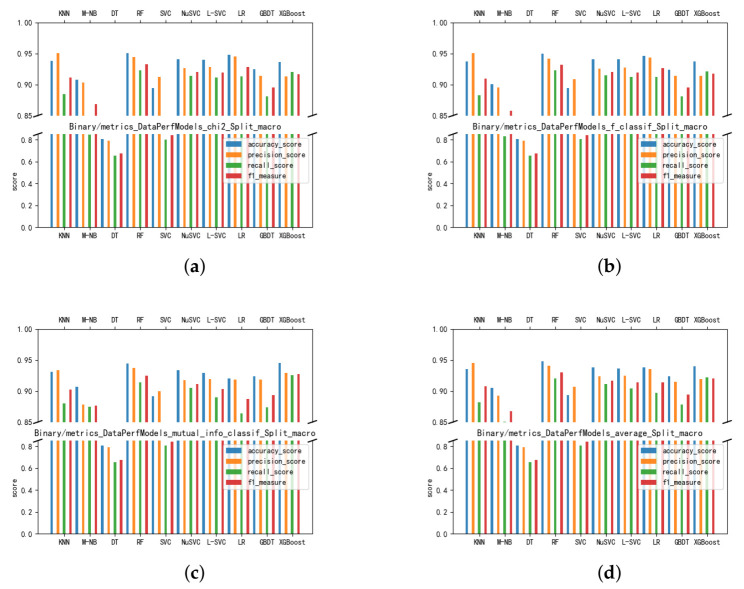
Models’ Performance on Candidate Feature Sets. (**a**) Chi-Squared Test. (**b**) ANOVA F-value. (**c**) Mutual Information. (**d**) Average Performance.

To select the optimal detection model and feature subset, we compared the average values of accuracy, precision, recall, and F1-measure of the candidate models on three feature subsets *chi2_784*, *f_784*, and *mutual_info_784* after 10-fold cross-validation. The candidate models we use are k-nearest neighbor (KNN), multinomial naive Bayesian (M-NB), decision tree (DT), random forest (RF), support vector classification (SVC), nuclear support vector classification (NuSVC), linear support vector classification (L-SVC), logistic regression (LR), gradient boosting decision tree (GBDT), and eXtreme gradient boosting (XGBoost). Figure 6 shows the performance of the models on each feature subset, where Figure 6a–c are the accuracy, precision, recall, and F1-measure on *chi2_784*, *f_784*, and *mutual_info_784*”, respectively, and Figure 6d is the average performance on the three feature subsets.

The experiments show that the random forest performs better on all three feature subsets and that the average performance is optimal, as seen in Figure 6d. Its average accuracy on the three feature subsets is 94.82%, and its average precision, average recall, and average F1-measure are 94.08%, 91.99%, and 92.97%, respectively. Among them, random forest has an overall advantage when comparing the average performance of the three feature subsets with XGBoost and NuSVC. Random forest significantly outperformed XGBoost in terms of accuracy, precision, recall, and F1-measure, with 93.96%, 91.89%, 92.23% and 92.04%, respectively. Random forest is significantly better than NuSVC in all four evaluation metrics, which showed a 93.82%, 92.33%, 91.12% and 91.70% performance in the four aspects. Finally, we identified the random forest as the static detection layer of the algorithm model.

Features selected using the chi-square test outperformed the other methods. Comparing the performance of the three feature subsets on random forests, the corresponding feature set of the chi-square test outperformed the other two methods on four measures. Figure 6a shows that the accuracy of random forest on the subset of *chi2_784* is 95.04%, and from Figure 6b,c, it can be concluded that *f_784*, *mutual_info_784* were 94.95% and 94.46%, respectively. In terms of the F1-measure, *chi2_784* achieved 93.28%, and *f_784* and *mutual_info_784* achieved 93.17% and 92.46%, respectively. Although the cardinality test does not differ significantly from the ANOVA f-value, it still presents a slight advantage.

The chi-squared test is a common hypothesis testing method, based on the chi-squared distribution, which belongs to the category of nonparametric tests and mainly compares the association between two categorical variables. The calculations are shown in Equation (11).

(11) $$\chi^{2}=\sum_{i=1}^{k}\frac{{\left(x_{i}-m_{i}\right)}^{2}}{m_{i}}$$

For a clearer representation of the feature selection results, Table 1 shows the top 20 features of the 2494 features selected after feature selection and their scores in order of importance. Among them, *android.intent.action.USER_PRESENT* is the most important, and is “a listener for unlocking”. The intent of this is explained in the official Android API Reference [35], as follows: “Broadcast Action: Sent when the user is present after the device wakes up (e.g., when the keyguard is gone). This is a protected intent that can only be sent by the system.” The cecond most important is *android.net.conn.CONNECTIVITY_CHANGE*, which is responsible for monitoring the network status of connections, and the third is *android.intent.action.VIEW*, which serves to display data to the user.

Of the top 20 most important features, five are SMS-related and, in addition to the common *send* and *receive* messages, the most notable is *<actionandroid:name= “android.provider.Telephony.SMS_RECEIVED”/>*, the intent commonly used by malware when implementing blocking SMS. There appear to be three network-related features, all of which can be used to change the network state of a device. In addition, *<actionandroid:name=“android.intent.action.BOOT_COMPLETED”/>* and *android.permission. RECEIVE_BOOT_COMPLETED*, which are logically required for the behavior to occur simultaneously, also exist in Table 1. These two features can monitor the boot-up of the phone and are used when an application intends to self-start from power-up.

Note that the *<actionandroid:name=“android.app.action. DEVICE_ADMIN_ENABLED”/>*, which appears in Table 1, in fifth place, is required if the application is to gain administrator privileges with the *BIND_DEVICE_ADMIN* at the same time; to some extent, this intent is very dangerous. However, the latter’s score was only 0.35, ranking 2651 in importance. According to statistics, there were 79 training samples that included only the former, none that included only the latter, and only one that included both features. Of the samples that contained only the first characteristic, there were six benign samples (7.6%) and 73 malicious samples (92.4%); Ransomware and Scareware accounted for 43.0% and 26.6% of the total, respectively. A detailed data comparison is shown in Table 3. The sample that contains both features is *com.gtp.nextlauncher.trial*; however, this sample is benign, which may mean that the malicious sample in the dataset dynamically requires *BIND_DEVICE_ADMIN* to gain admin access to the device. Therefore, to some extent, dynamic analysis is necessary.

After the above, the detection algorithm for the static detection layer was implemented with random forest, with a detection rate of 95.04%. Although lower than the 95.22% of [20], our method uses only 784 static features, which are only one-tenth of the 8115 features used in [20]. This was acceptable to us despite the reduced detection rate.

### 4.5. Res7LSTM Performance Comparison

The malicious samples in the output of the static detection layer and the benign samples with corresponding probabilities below the threshold are the inputs used in this layer. The current classification layer uses the Res7LSTM model algorithm to perform three classification tasks on the dynamic network traffic characteristics of the input samples. Detailed experiments will be presented later.

**Figure 7:**
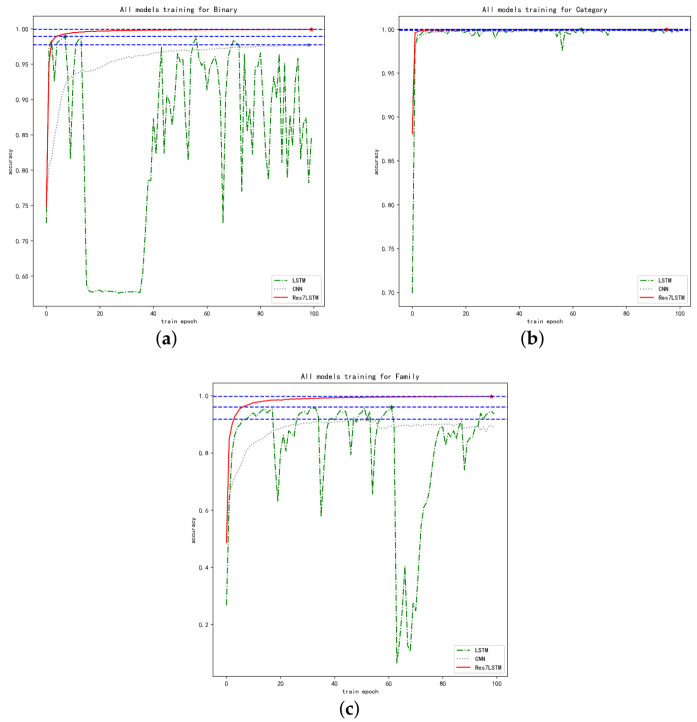
Comparison of the accuracy of CNN, LSTM and Res7LSTM. (**a**) Binary classification. (**b**) Category classification. (**c**) Family classification.

Figure 7 is a comparison of the training accuracy of LSTM, CNN, and Res7LSTM when applied to the three classification tasks, where Figure 7a shows the changes in the training accuracy of the three models when performing the binary classification task, and Figure 7b,c show the accuracy trends for malware category classification and malware family classification, respectively.

The training accuracy changes of the CNN, LSTM and Res7LSTM models are compared in three classification tasks. Res7LSTM exhibited an excellent performance in both binary classification and family classification, as shown in Figure 7a,c. Although the latter two performed similarly, LSTM did not perform steadily in this experiment, and CNN was the worst of the binary classification tasks. Figure 7b shows that all three models exhibited good performance for malware category classification. Our model Res7LSTM outperformed the first two models on all three tasks and achieved an accuracy of 99.98% on binary classification. On the four-category task, i.e., category classification, all three models achieved 99.99% accuracy. For 40-category classifications, i.e., family classification, Res7LSTM achieved 99.91% precision and 99.93% recall, whereas CNN only achieved 94.50% and 93.04%.

**Figure 8:**
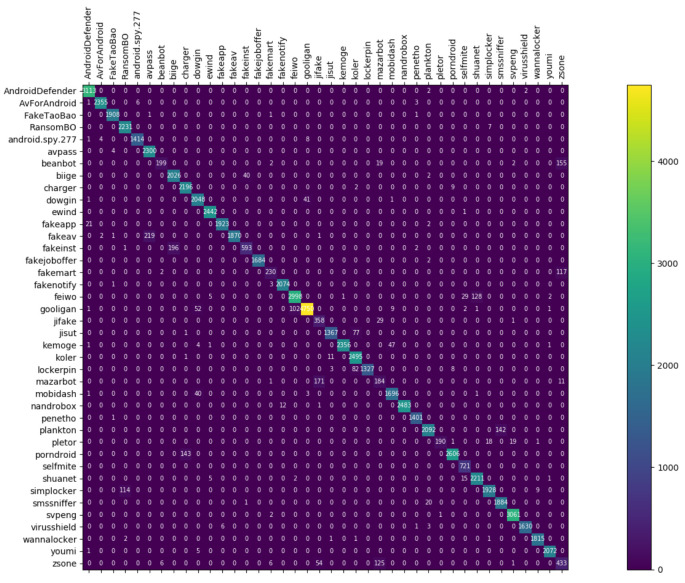
Confusion Matrix for CNN.

**Figure 9:**
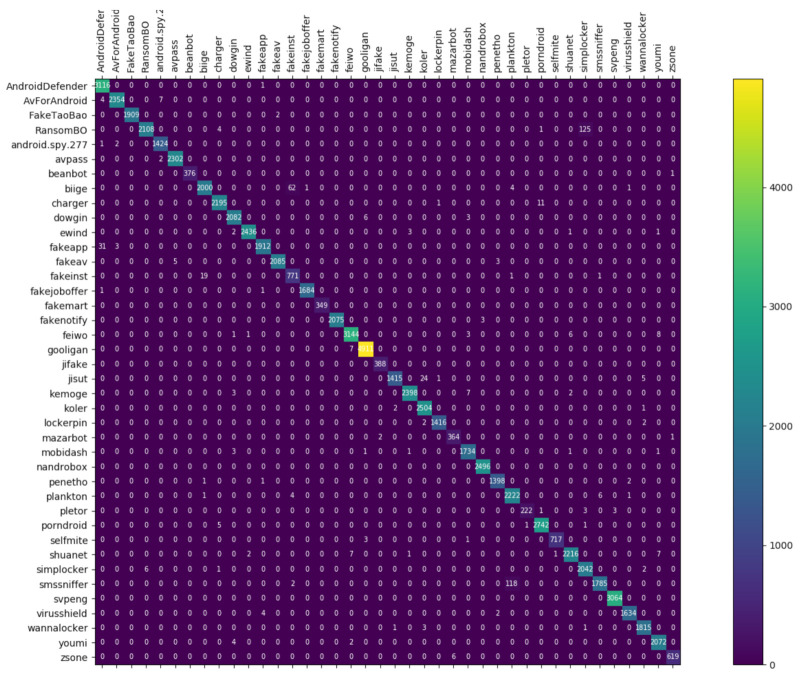
Confusion Matrix for LSTM.

**Figure 10:**
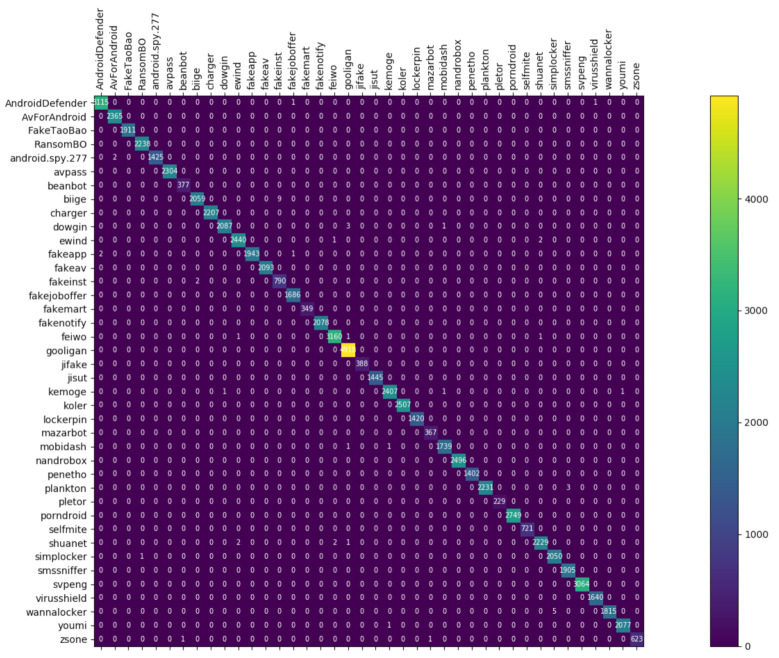
Confusion Matrix for Res7LSTM.

**Figure 11:**
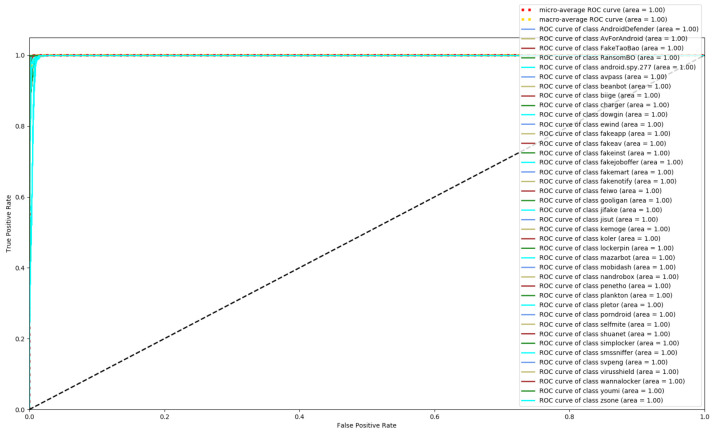
ROC for CNN.

**Figure 12:**
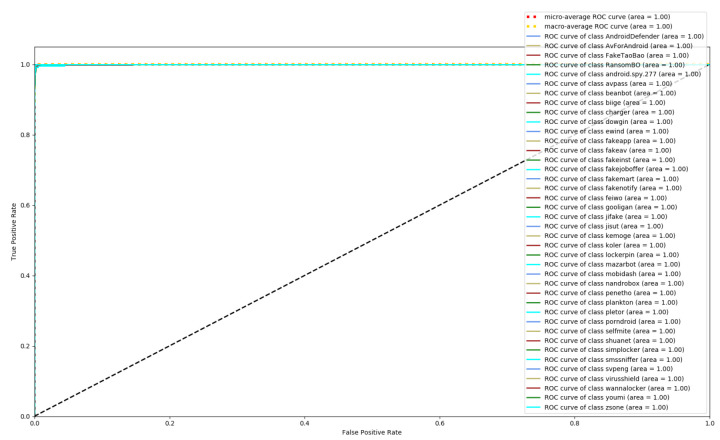
ROC for LSTM.

**Figure 13:**
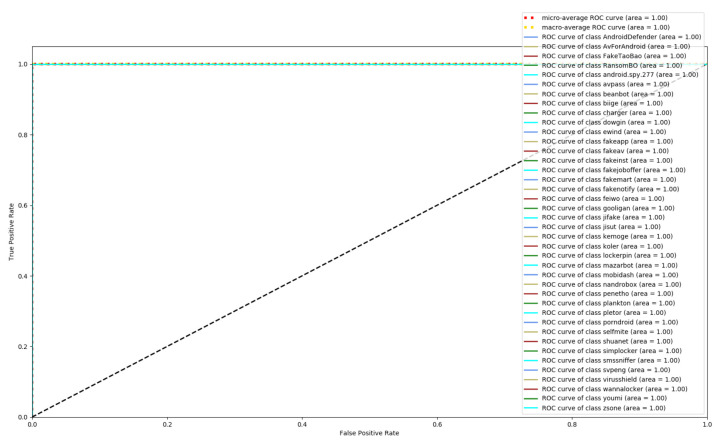
ROC for Res7LSTM.

Figure 8, Figure 9 and Figure 10 depict the confusion matrices of the three models in malware family classification. Figure 8 shows the confusion matrix of CNN, which is less effective in classifying beanbot, jifake, mazarbot, and zsone families, where the precision rate of mazarbot is only 50.54%(184 samples were correctly predicted and 183 were incorrectly predicted) and that of beanbot is only 52.79% (199 samples were correctly predicted and 178 were incorrectly predicted) and 65.90% (230 samples were correctly predicted and 119 were incorrectly predicted) on fakemart. It should be noted that all three families belong to the same SMS category, which could means that although CNN learns the differences between categories very well, it is still difficult to detect the differences within the malware families. Figure 9 shows the results of the LSTM on the test set, which is significantly better than the CNN, but regrettably the model does not work as well on the smssniffer, with a precision rate of 93.70%; in addition, the family RansomBO has a precision rate of only 94.13%. The confusion matrix is given in Figure 10 for our model, on a test sample of 75,099 traffic grayscale maps. Plotting from the True Positive Rate (TPR) and False Positive Rate (FPR) yields Receiver Operating Characteristic (ROC) curve. Figure 11, Figure 12 and Figure 13 show the ROC curves for each of the three models, and iy can be seen in Figure 13 that the curves are closer to the (0, 1) coordinate point compared to the others. It was experimentally demonstrated that our approach overcomes the shortcomings of the previous two models and exhibits an excellent performance on family classification.

**Figure 14:**
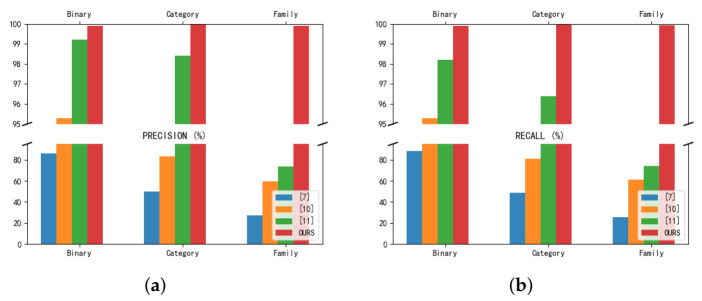
Res7LSTM vs. [16,17,20]. (**a**) Comparison of Precision. (**b**) Comparison of Recall.

To compare the performance difference between our method and other methods, refs. [16,17,20], which also use the CICAndMal2017 dataset, are used as comparators, and the comparison results, in terms of precision rate and recall, are shown in Figure 14. The precision rates for the binary classification, malware category classification, and malware family classification were 85.8%, 49.9%, and 27.5% in [16], 95.3%, 83.3%, and 59.7% in [17], and 99.2%, 98.4%, and 73.5% in [20], respectively. Our method achieved 99.98% accuracy in binary classification, 99.99% accuracy in malware category classification, and increased accuracy, to 99.6%, in malware family classification. In terms of recall, the classification performance for 2, 4 and 40 categories was 88.3%, 48.5% and 25.5% in [16], 95.3%, 81%, and 61.2% in [17] and 98.2%, 96.4%, and 74.2% in [20], respectively. Our approach achieves a 99.98% recall rate in the binary classification task and improves the malware category classification and malware family classification to 99.99% and 99.93%, respectively. After this comparison, our method solves the difficulties and problems faced by the previous methods in malware family classification.

## 5. Conclusions and Limitations

This study proposes a hybrid analysis-based process for Android malware detection and classification, improving both static features and dynamic network traffic features. In the detection layer, permissions and intent are used as input static features, and after feature selection and a comparison of their performance on different algorithms, the optimal static detection algorithm and the optimal feature selection method are determined, namely, random forest and chi-square test. A final detection rate of 95.04% was obtained. Despite the loss of the 0.18% detection rate, we used very few features and found that there were many irrelevant and redundant features in the original features through experimentation. At the end of the static test, we analyzed some important features. To further detect low-trust benign samples and detected malware, a dynamic analysis layer will detect and classify these samples. In the dynamic analysis layer, images of the network traffic generated by the application software during dynamic execution were further classified and detected using Res7LSTM. After different classification experiments, our method shows excellenct abilities for malware detection and further subclassification, among which the Android malware category and family classification were much improved compared to other methods, especially in terms of family classification. Overall, the combination of static detection and an analysis of dynamically generated network traffic can effectively solve the problem of low-trust benign samples that are not detected correctly while, at the same time, helping to classify malware families.

However, there are still some problems with our work. The code obfuscation and encryption were not considered in the static detection process, i.e., no validity check was performed on the obfuscated and encrypted samples, which may not be sufficient for such malware detection. Code obfuscation and encryption are a major threat to the validity of the results of this experiment. In the dynamic analysis, the dynamic analysis features used are relatively singlular, using only traffic files and ignoring other features such as memory logs, API operation logs, and device information logs, which may not be sufficient to carry out an effective detection for malware that is not primarily network-based. This, likewise, has a significant impact on the validity of the experiment. In addition, dynamic analysis methods using flow images are less interpretable. These issues will be the focus of our future work.

## Figures and Tables

**Table 1:** The Top 20 Important Features and One Interesting Feature.

Feature	Score
<actionandroid:name=“android.intent.action.USER_PRESENT”/>	316.54
<actionandroid:name=“android.net.conn.CONNECTIVITY_CHANGE”/>	259.02
<actionandroid:name=“android.intent.action.VIEW”/>	243.52
android.permission.SYSTEM_ALERT_WINDOW	225.62
<actionandroid:name=“android.app.action.DEVICE_ADMIN_ENABLED”/>	185.42
android.permission.READ_PHONE_STATE	185.31
android.permission.SEND_SMS	175.83
<actionandroid:name=“android.intent.action.PACKAGE_ADDED”/>	175.34
android.permission.CHANGE_NETWORK_STATE	150.87
android.permission.RECEIVE_SMS	150.55
android.permission.MOUNT_UNMOUNT_FILESYSTEMS	140.80
<actionandroid:name=“android.provider.Telephony.SMS_RECEIVED”/>	138.15
android.permission.GET_TASKS	136.95
<actionandroid:name=“android.intent.action.BOOT_COMPLETED”/>	124.74
android.permission.READ_SMS	123.28
android.permission.RECEIVE_BOOT_COMPLETED	122.16
android.permission.CHANGE_WIFI_STATE	113.41
android.permission.WRITE_SMS	106.71
<actionandroid:name=“android.intent.action.MAIN”/>	104.34
<actionandroid:name=“.ACTION_DECABDCE”/>	103.92
……	……
<actionandroid:name=“android.permission.BIND_DEVICE_ADMIN"/>	0.35

**Table 2:** Data set distribution and flow file processing results.

Binary	Category	Family	Samples	Number of Traffic Pcap/Png		
				Train	Test	Total
**Benign**	**None**	**None**	**1190**	**393,364**	**44,418**	**437,782**
Malware	Adware	dowgin	10	18,785	2091	20,876
		ewind	10	21,950	2443	24,393
		feiwo	15	28,390	3163	31,553
		gooligan	14	44,211	4918	49,129
		kemoge	11	21,663	2410	24,073
		mobidash	10	15,618	1741	17,359
		selfmite	4	6472	721	7193
		shuanet	10	20,060	2234	22,294
		youmi	10	18,650	2078	20,728
		**TOTAL**	**94**	**195,799**	**21,799**	**217,598**
	Ransomware	charger	10	19,807	2207	22,014
		jisut	10	12,951	1445	14,396
		koler	10	22,516	2507	25,023
		lockerpin	10	12,732	1420	14,152
		pletor	10	2027	229	2256
		porndroid	10	24,692	2749	27,441
		RansomBO	10	20,090	2238	22,328
		simplocker	10	18,394	2051	20,445
		svpeng	11	27,512	3064	30,576
		wannalocker	10	16,352	1820	18,172
		**TOTAL**	**101**	**177,073**	**19,730**	**196,803**
	Scareware	android.spy.277	6	12,815	1427	14,242
		AndroidDefender	17	27,976	3117	31,093
		AvForAndroid	10	21,246	2365	23,611
		avpass	10	20,695	2304	22,999
		fakeapp	10	17,469	1946	19,415
		fakeav	9	18,790	2093	20,883
		fakejoboffer	9	15,124	1686	16,810
		FakeTaoBao	9	17,171	1911	19,082
		penetho	10	12,576	1402	13,978
		virusshield	10	14,728	1640	16,368
		**TOTAL**	**100**	**178,590**	**19,891**	**198,481**
	SMS	beanbot	9	3349	377	3726
		biige	11	18,563	2068	20,631
		fakeinst	10	7080	792	7872
		fakemart	10	3095	349	3444
		fakenotify	10	18,653	2078	20,731
		jifake	10	3442	388	3830
		mazarbot	9	3267	367	3634
		nandrobox	11	22,426	2496	24,922
		plankton	10	20,065	2234	22,299
		smssniffer	9	17,102	1905	19,007
		zsone	10	5587	625	6212
		**TOTAL**	**109**	**122,629**	**13,679**	**136,308**
	**TOTAL**	**NaN**	**404**	**674,091**	**75,099**	**749,190**
**TOTAL**	**NaN**	**NaN**	**1594**	**1,067,455**	**119,517**	**1,186,972**

**Table 3:** Distribution of DEVICE_ADMIN_ENABLED features in the sample.

Binary	Category	Samples	%
Malware	Ransomware	34	43.0
	Scareware	21	26.6
	Adware	12	15.2
	SMS	6	7.6
Begin	None	6	7.6

## Data Availability

Not applicable.

## Abbreviations

The following abbreviations are used in this manuscript:

SMS	Short Message Service
NuSVC	Nuclear Support Vector Classification
ResNet	Residual Neural Network
RNN	Recurrent Neural Network
LSTM	Long short-term Memory Network
RF	Random Forest
KNN	k-nearest neighbor
GBDT	Gradient Boosting Decision Tree
XGBoost	eXtreme Gradient Boosting
TP	True Positives
TN	True Negatives
FP	False Positives
FN	False Negatives
